# Fluorescence-guided fiber-optic micronavigation using microscopic identification of vascular boundary of liver segment and tumors

**DOI:** 10.7150/thno.45973

**Published:** 2020-05-15

**Authors:** Qingliang Wang, Baifeng Qian, Michael Schäfer, Wolfgang Groß, Arianeb Mehrabi, Eduard Ryschich

**Affiliations:** 1 Department of General, Visceral and Transplantation Surgery, University Hospital Heidelberg, Heidelberg, Germany; 2 Department of General Surgery, The Third Affiliated Hospital of Sun Yat-sen University, Guangzhou, China

**Keywords:** Vascular boundary, Fluorescence imaging, Endothelial capture, Surgical micronavigation, Fiber-optic microscopy

## Abstract

**Background**: The exact identification of tumor boundaries and related liver segments is especially important for liver tumor surgery. This study aimed to evaluate a new approach for vascular boundary assessment and surgical navigation based on fiber-optic microscopy and microvascular fluorescence labeling.

**Methods**: Antibody clones with fast binding ability were identified and selected using immunofluorescence. We evaluated the endothelial capture efficacy for an anti-mouse CD31 antibody labeled with different fluorophores and different degrees of labeling *ex vivo*. Segment boundary identification and navigation potential using endothelial capture were explored by two different fiber-optic microscopy systems. Finally, microvasculature labeling and fiber-optic microscopy were used to identify and treat microscopic liver tumors *in vivo*.

**Results**: The following monoclonal antibodies were selected: anti-mouse CD31 (clone 390), anti-mouse CD54 (YN1/1.7.4), anti-human CD31 (WM59), and anti-human CD54 (HA58). These clones showed fast binding to endothelial cells and had long half-lives. The fluorophore choice and the degree of antibody labeling did not significantly affect capture efficacy in an isolated liver perfusion model. The microvascular system was clearly identified with wide-field fiber-optic microscopy after labeling the endothelium with low doses of specific antibodies, and the specifically labeled liver segment could be microscopically dissected. High antibody doses were required for confocal laser endomicroscopy. After microscopically identifying the vascular margin *in vivo*, tumor thermoablation strongly reduced tumor size or totally eliminated tumors.

**Conclusions**: We demonstrated that vascular boundaries of liver tumors and locally perfused liver segments were accurately identified and surgical micronavigation was facilitated with fiber-optic microscopy and selected endothelium-specific antibodies.

## Introduction

Anatomic liver resection is one of the most frequent surgical operations which is used for the treatment of liver tumor, such as hepatocellular carcinoma (HCC), and metastases of gastroenterological cancers 1, 2. During this operation, the accurate determination of liver segment anatomy is necessary to perform surgery without complications and ensure long-term outcomes 3, 4. Several methods are currently used for intraoperative liver segment identification. First, ultrasound is widely applied for intraoperative navigation in liver surgery 5, 6. Additionally, liver segment visualization using superselective injection of indigo carmine 7, and indocyanine green (ICG) 8, 9 has been intensively studied to label liver segments. The use of indigo carmine is quite disadvantageous because the segment demarcation is not exact, the labeling is very short and disappears 10 min after injection 10.

ICG has some application-specific disadvantages that prevent its wide application to surgical navigation settings 11. Although ICG is effective for liver segment labeling, it does not always provide a sufficient segment contrast and temporal stability because the dye disappears gradually after injection 12, 13. ICG injection is only possible under ultrasound control, but the surgeon must be well trained in ultrasound techniques. The use of ultrasound may also be difficult in some cases such as repeated liver resection. ICG is also contradicted in patients with iodine allergy 14. Alternative technologies that result in better labeling and in stronger demarcation of segment boundaries may improve intraoperative navigation in liver surgery.

ICG is also used for direct tumor labeling. For this aim, ICG has been systemically injected. 2-5 days after application, ICG is retained in hepatic tumors; thus, it is used clinically to improve intraoperative tumor identification 11, 15. Although this method has a growing popularity in surgery, it is accompanied by incomplete tumor labeling 16, and by relatively high rates of false-positive and false-negative results 17.

Experimental and clinical studies have identified alternative tools for image-guided surgical navigation, such as fluorescence-labeled antibodies 18-20. Upon systemic administration, circulating antibodies are in direct contact with endothelial cells, and they immediately bind to specific antigens. This fluorescence technique has shown promise for imaging selected liver segments both macro- and microscopically 21, 22.

Furthermore, antibody-based fluorescence imaging is useful for targeting tumors. Previous studies were mainly based on tumor-specific antibody uptake 20, and they showed a rather high degree of sensitivity and specificity for gross local tumor identification 23. However, it is important to delineate the exact tumor margins, particularly for achieving R0 tumor resections. The accuracy of existing methods is limited by inadequate spatial resolution.

A recent study described the unique phenomenon of antibody capture by the endothelium (endocapt) 22. After a locoregional antibody injection, endocapt leads to site-specific antibody accumulation on the endothelium 22. The other study showed that fluorescent ramucirumab endocapt enabled excellent fluorescence imaging of selected liver segments in a preclinical model 21.

In addition to macroscopic fluorescence imaging, fiber-optic endomicroscopy represents a new promising technique for identifying morphological structures at the cellular level. Currently, some clinical applications are based on confocal laser endomicroscopy (CLE), including *in situ* diagnoses of Barrett-esophagus 24, 25, cystic diseases 26, and colorectal lesions 27, and CLE-guided needle biopsies 28.

In the current study, we examined the feasibility of fiber-optic microscopy (FOM) and endothelium-specific fluorescent labeling in the microvascular system for surgical micronavigation and vascular boundary identification.

## Materials and methods

### Histological staining and effective antibody concentrations

Human samples were provided by the tissue bank of the National Center of Tumor Diseases (Heidelberg, Germany) and by PancoBank of the European Pancreatic Center (University Hospital Heidelberg). All samples were used in accordance with the regulations of both tissue banks and the Ethics Committee of the University of Heidelberg.

Frozen sections were stained for 15 min or 10 s by either direct or indirect immunofluorescence staining. The principle for choice of incubation time (10 s, 15 min) was previously described 22. For direct 1-step immunofluorescence, R-Phycoerythrin (RPE)-conjugated anti-mouse antibodies were used. Indirect 2-steps staining was performed using non-labeled primary antibody followed by 1 μg/mL of RPE-conjugated secondary antibody. The respective antibodies are listed in Table 1. The endothelium-bound fluorescent antibodies were then visualized using fluorescence microscopy (Axio Observer.Z1, Zeiss, Jena, Germany) equipped with monochromatic LED light sources (Colibri, Zeiss) with peak excitation wavelength of 470 nm (for fluorescein and Alexa Fluor (AF) 488), 555 nm (for RPE), 625 nm (for AF649) and multispectral filter set (90HE, Zeiss). All images were processed using ZEN software (ZEN 2.3, Zeiss). The mean fluorescence intensity (MFI) of labeled blood vessels was measured on each tissue slide, and the value was corrected for the background signal. The binding characteristics of selected antibody clones were quantitatively evaluated and expressed as the half-maximal effective concentration (EC_50_) as previously described 22, 29. To calculate the EC_50_ value, the MFI values were further analyzed with the customized SCTMult software (version 1.3.0.1, W. Groß). For calculation, the non-linear regression fit to the Hill equation of this software was used. Any linearization methods like Scatchard or Lineweaver-Burk were not used.

Vascular expression of CD34 in paraffin-embedded human liver tumors was analyzed with an immunohistochemistry kit (ZytoChem Plus AP Polymer Kit, Zytomed, Berlin, Germany) according to manufacturer instructions. The tumor samples were derived from 48 patients with hepatocellular carcinoma (HCC), 20 patients with liver metastases from colorectal cancer (Crc MTS), and 17 patients with liver metastases from pancreatic cancer (LMTS).

### Cell culture

Murine cell lines NIH/3T3, Hep55.1C and Panc02 (CLS, Heidelberg, Germany), were grown in Iscove's DMEM medium supplemented with 10% heat-inactivated fetal calf serum, penicillin, streptomycin, and L-glutamine (c-c-pro, Oberdorla, Germany). Human endothelial cell lines (passage 7-10), human umbilical vein endothelial cells (HUVEC,) and human dermal microvascular endothelial cells (HDMEC) (PromoCell, Heidelberg, Germany), were cultured in endothelial cell growth medium and endothelial cell growth medium MV2 (PromoCell) respectively. The murine endothelial cell line, bEnd.3, was purchased from ATCC (Manassas, VA, USA) and cultured in the recommended medium, DMEM. All cell lines were cultured in a humidified incubator at 37°C with 5% CO2.

### Cell-based antibody binding and toxicity* in vitro*

NIH/3T3 (1 × 10^4^/ channel) and bEnd.3 (8 × 10^3^/ channel) cells were seeded into IV-*μ* Ibidi microfluidic chambers (Ibidi, Martinsried, Germany) and incubated for 24 h. To achieve high CD54 (intercellular adhesion molecule-1, ICAM-1) expression levels, bEnd.3 cells were treated for 16 h with 100 ng/mL recombinant murine TNF-α (ImmunoTools, Friesoythe, Germany). Viable cells were then stained for 10 s or 15 min with R-Phycoerythrin (RPE)-conjugated clone 390 (1 µg/mL), YN1/1.7.4 (1 µg/mL), or HM34 (2 µg/mL) antibodies. After a 15-min incubation, the selected clones were quantitatively analyzed by calculating the EC_50_ as described above. The time-course of the fluorescence signal change was analyzed to evaluate the intracellular metabolism of antibodies. After staining for 15 min, the medium was replaced with fresh medium, and at 1, 2, 4, and 24 h, the fluorescence intensity was recorded. The half-life time of antibody retention in living cells was calculated using fluorescence signal as previously described 22, 29.

HUVEC and HDMEC cells (1.5 × 10^4^/ well) were cultured in 48-well plates (Greiner Bio-One, Frickenhausen, Germany) and treated for 16 h with 10 ng/mL TNF-α (PeproTech, Rocky Hill, NJ, USA) to characterize the binding of antibody clone HA58. Antibody uptake and retention were analyzed as described above at 3, 6, 24, and 48 h. Resazurin cell viability assay (R&D Systems, Minneapolis, MN, USA) was used to analyze the cytotoxicity after antibody binding according to the manufacturer's instructions.

### Antibody labeling

Fluorescein isothiocyanate (FITC, Sigma-Aldrich, Deisenhofen, Germany) was conjugated to anti-CD31 antibody (clone 390) at varying ratios of fluorophore/protein (F:P). After labeling, the concentration of protein was determined by Pierce BCA Protein Assay Kit (Thermo, Waltham, MA, USA) and the concentration of fluorescein was measured by fluorimetry (FluoStar Optima, BMG Labtech, Ortenberg, Germany). The molar F:P was calculated to represent the degree of labeling (DOL). Other antibodies were labeled with Alexa Fluor (AF647 NHS Ester; Thermo), according to manufacturer's instructions, except the incubation time was adjusted to 2 h to achieve higher DOLs.

### Endothelial antibody capture* ex vivo*

The livers of male C57BL/6 mice (Charles River, Sulzfeld, Germany) were isolated and perfused through the portal vein as previously described 22. For liver segment perfusion, the left hepatic pedicle and the vessels that supplied the omental segment were ligated with microclips. All branches were clamped, except the one that supplied the left posterior segment. For the purpose of liver subsegment labeling, 400 ng (50 μL) of RPE-conjugated antibody clone 390 was injected, followed by perfusion with 0.4 mL modified Krebs-Henseleit buffer (Sigma-Aldrich, Taufkirchen, Germany) at a flow rate of 0.1 mL/min. The flushed solution was collected, and the concentration of unbound fluorescent antibody was determined with fluorimetry (FluoStar Optima). The concentration of unlabeled antibody was determined with an enzyme-linked immunosorbent assay (ELISA, Rat IgG ELISA Kit, Thermo). The capture efficacy was calculated as the percentage of antibody captured in the liver, and the endothelium-bound antibody was visualized with microscopy.

### Fiber-optic microscopy

CLE was performed with a commercial, probe-based endomicroscope (Cellvizio, Mauna Kea Technologies, Paris, France) equipped with a ProFlex^TM^ S 1500 imaging probe, with a 1.5 mm diameter, a 600-µm field of view, and a lateral resolution of 3.3 µm.

The wide-field FOM (WF-FOM) was assembled (Figure 5A) according to a previously described construction method 30. All optomechanics except the objective (EC Plan-Neofluar 10×/0,3 Ph1, Zeiss), were purchased from Thorlabs (Newton, NJ, USA). For specific detection of RPE fluorescence, following light source and optics were used in the construction: LED source (MINTL5) with peak wave length of 554 nm; excitation filter FB530-10, emission filter FB580-10, dicroic mirror DMLP550 (all from Thorlabs). The fiber-optic bundle (Grintech, Jena, Germany) had 30,000 single fibers, a 790-µm field of view, and 1-m length. Monochromatic cameras (Kiralux SC505MU, Thorlabs; or DX4-285, Kappa, Gleichen, Germany) were used for imaging.

### Mouse tumor model and endothelial capture-guided therapy* in vivo*

Mouse hepatic primary and metastatic tumor models were induced by inoculation of Hep55.1C and Panc02 cells. Briefly, tumor cell suspension (1.2 to 1.6 × 10^6^ cells) was injected into the defined liver segments (left anterior or posterior segment) with a 20-µL syringe (Hamilton, Bonaduz, Switzerland). Subsequent experiments were performed 12-14 days after the injections. For *in vivo* labeling, tumor-bearing mice were anesthetized, and 5 µg of RPE-conjugated anti-CD31 antibody (clone 390) was selectively injected into the hepatic artery using a 25-µL syringe fitted with a 34-G needle (Hamilton) as previously described 22.

Another micro-metastatic mouse model was generated with intrahepatic subcapsular injections of Panc02 cells (2 to 4 × 10^5^) resuspended in 1-2 µL of PBS. Mice were anesthetized again 5 days after tumor cell inoculation, and a median laparotomy was performed. The immediate labeling of micro-vascular system was achieved after intravenous injection of 15 µg of RPE-conjugated anti-CD31 antibody (clone 390). Microscopic tumors (1-2 mm) and vascular tumor boundaries were identified according to the clear difference of micro-angioarchitecture between tumor and normal liver. Tumor-bearing mice were then randomly assigned to an experimental (therapy) or control group. Image-guided local thermal ablation was accomplished using needle electrocautery (Erbotom T300C, Erbe Elektromedizin, Tübingen, Germany) in the experimental group while no treatment was done for the control group. All liver samples were collected at the end of the experiment and inspected pathologically. Assuming an elliptical tumor form, tumor volumes were calculated with the formula: 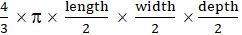.

### Statistical analysis

Statistical analysis was performed with SPSS 22.0 (IBM, Armonk, NY, USA). Data are presented as the mean ± SD. To analyze differences between the groups, a t-test, one-way ANOVA with *post hoc* Bonferroni correction or Kruskal-Wallis test was used. *P* values <0.05 were considered statistically significant.

## Results

### Endothelium-specific antibody binding to human and mouse tissues

**Figure 1:**
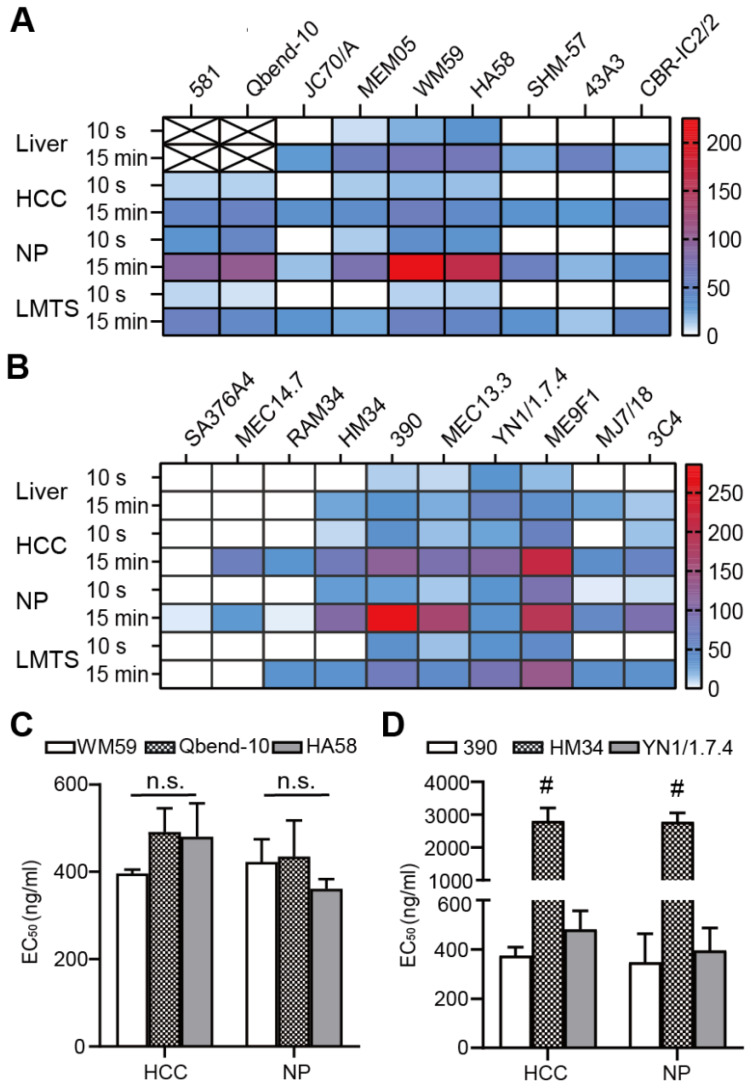
Quantitative evaluation of the binding characteristics of different endothelium-specific antibodies in human and mouse tissues. (A, B) The mean fluorescence intensity (color encoded) of each antibody clone is compared between human tissue (A) stained indirectly and mouse tissue (B) stained directly with immunofluorescent antibodies for 10 s or 15 min (n=2-3). Cross-marked fields indicate that only large blood vessels were labeled in human liver. (C, D) Comparison of EC_50_ values for the selected antibody clones in human (C) and mouse tissue (D) after incubating for 15 min (n=2). HCC: hepatocellular carcinoma; NP: normal pancreas; LMTS: liver metastasis of pancreatic cancer; n.s. no significant difference;^ #^ *P*<0.01.

The initial identification of fast-binding antibodies was performed using immunofluorescence staining. After a 15-min incubation, all antibodies showed detectable, ubiquitous endothelial binding in human (Figure S1A-B) and mouse (Figure S1C-D) tissue samples. There was no detectable binding for all the RPE-labeled isotypic antibodies. In human liver, the anti-CD34 monoclonal antibody (mAb) only labeled large blood vessels, not liver sinusoids (Figure S1A). The majority of antibodies failed to bind during the short incubation time. Those that bound to the endothelium in human tissues were: anti-CD34 antibodies (clones Qbend-10, 581), anti-CD31 antibodies (clones WM59, MEM05) and anti-CD54 antibodies (HA58). Those that bound to the endothelium in mouse tissues were: anti-CD31 antibodies (clones 390, MEC13.3), anti-CD54 antibody (YN1/1.7.4), and anti-CD146 antibody (ME9F1). The MFIs were displayed in a heat map, where “zero” (white color code) indicates no binding (Figure 1A-B). The anti-CD31 and anti-CD54 mAbs showed the best binding properties, and were selected for subsequent experiments. Considering its nearly perfect specific expression in human tumor tissues, the anti-CD34 mAb was also considered a potential candidate. To quantify the binding potential, the EC_50_ value of selected antibody clones was measured in HCC and pancreas tissues after staining for 15 min. EC_50_ values varied from 396 to 491 ng/mL in human HCC tissues, and 361 to 435 ng/mL in human normal pancreas tissues, with no significant differences between the selected clones (Figure 1C). In mice, clone HM34 had a significantly higher EC_50_ value than the other two antibody clones (Figure 1D). The EC_50_ values for clone 390 and YN1/1.7.4 were not significantly different (Figure 1D).

### Antibody binding and metabolism *in vitro*

**Figure 2:**
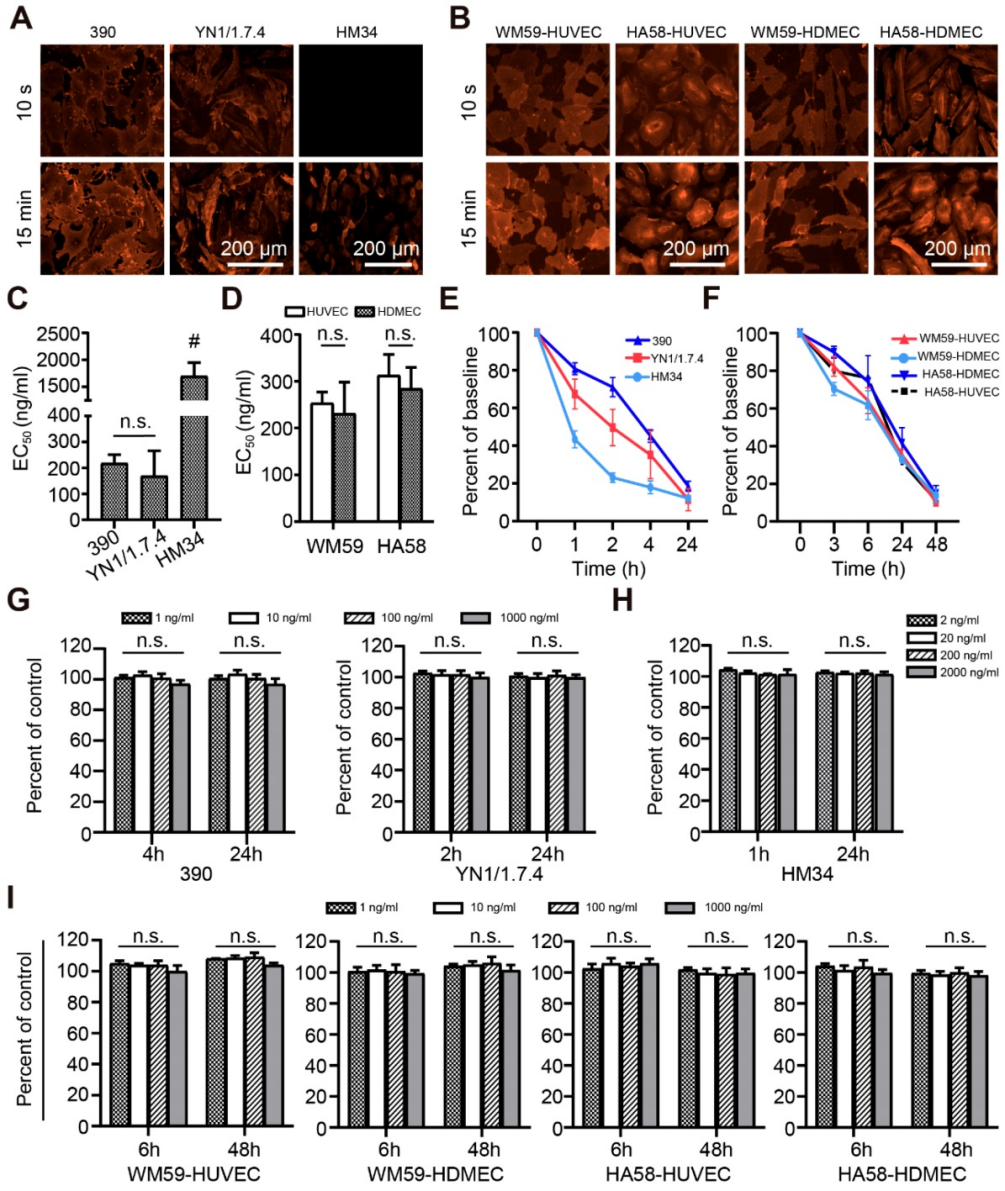
Antibody binding and metabolism in living cells *in vitro*. Representative immunofluorescence images show the binding of selected anti-mouse (A) and anti-human (B) mAb clones to living endothelial cells. (C, D) EC_50_ values of anti-mouse (C) and anti-human (D) mAb clones after binding to living cells for 15 min, n=3. (E, F) Quantitative assessment of the disappearance (due to capture, uptake, and elimination) of anti-mouse (E) and anti-human (F) mAb clones in cell cultures, n=3. (G-I) Cytotoxic effects on living cells *in vitro*, after different incubation times for anti-mouse mAbs at concentrations of 1-1000 ng/mL (G) or 1-2000 ng/mL (H), or (I) anti-human mAbs at 1-1000 ng/mL, n=3. n.s. no significant difference. mAb: monoclonal antibody; HUVEC: human umbilical vein endothelial cells; HDMEC: human dermal microvascular cells;^ #^ *P*<0.01.

The binding characteristics of selected antibody clones to living cells was analyzed *in vitro*. There was no detectable binding of isotypic antibody. No fluorescent signal was detected for clone HM34 after a short contact time, but binding was observed after a longer incubation (Figure 2A). Antibody clones 390, YN1/1.7.4, WM59, and HA58 rapidly bound to endothelial cell membranes (Figure 2A-B). Clone HM34 had a significantly higher EC_50_ value than the other mouse clones (Figure 2C). The EC_50_ values of all other clones were low, with no significant difference between cell lines (Figure 2C-D).

To evaluate the stability of the fluorescent signal, we microscopically observed the uptake and internalization of membrane-bound antibodies. After antibodies were taken up into the cytoplasm, the detectable fluorescence decreased with a half-life of 1-12 h depending on the clone (Figure 2E-F). Cell viability did not significantly change after treating with different doses of antibodies for different time intervals (Figure 2G-I).

### Antibody labeling and endocapt* ex vivo*

**Figure 3:**
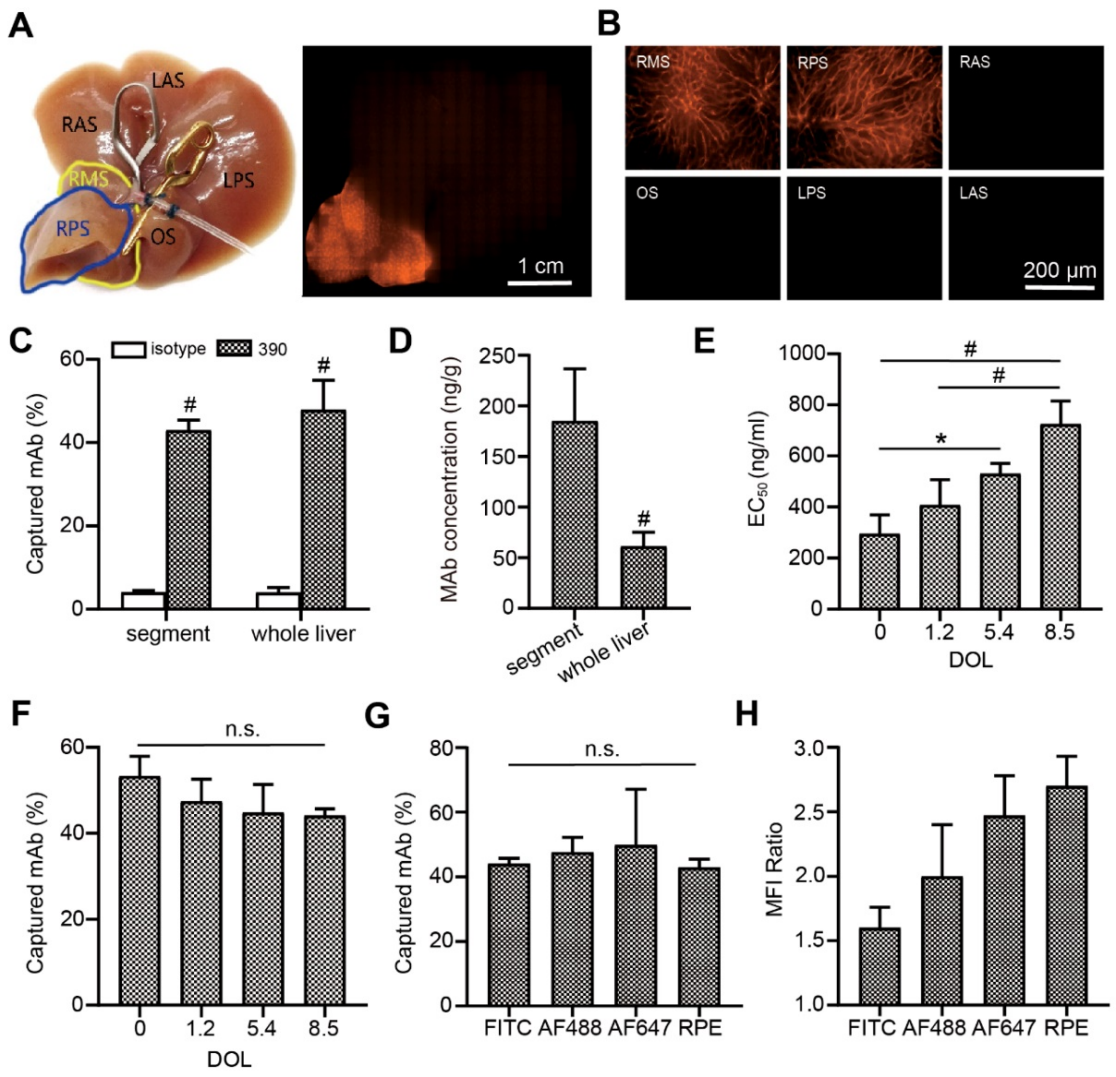
Endothelial antibody capture in isolated perfused mouse livers, *ex vivo*. (A, B) Representative macroscopic (A) and microscopic (B) images of fluorescence microscopy after segment perfusion. Segments (S) are labeled RA, LA: right and left anterior; RP, LP: right and left posterior; RM: right middle; O: omental. (C) Antibody capture efficacy and tissue concentrations (D) after perfusion either the whole liver or a liver segment with 200 ng RPE-labeled clone 390 mAb, n=3. (E) EC_50_ values for clone 390 at different degrees of labeling (DOL: fluorophore/protein ratio) in mouse pancreas. (F) Antibody capture efficacy in segments perfused with 200 ng of antibody at different DOLs; n=3. (G) Antibody capture efficacy after segments were perfused with 200 ng of four different fluorophore-labeled antibodies: AF488, RPE, high degree of FITC (DOL: 8.5), and high degree of AF647 (DOL: 9.3); n=3-4. (H) Mean fluorescence intensity (MFI) ratios indicate local antibody enrichment and imaging contrast for different fluorophore-labeled antibodies. n.s. no significant difference; ^*^ *P*<0.05. ^#^ *P*<0.01.

To evaluate the capture efficacy of endothelium-specific antibodies, RPE-labeled clone 390 mAb was perfused with an isolated mouse liver model. Macroscopic imaging showed that the perfused segment sharply contrasted with the other segments (Figure 3A). Microscopic fluorescence imaging revealed that labeling was excellent in the microvascular system (Figure 3B). The capture efficacies were 47.9% for the whole liver, and 43.0% for the perfused segment. In contrast, the capture of the corresponding isotypic antibody was nearly zero (Figure 3C). The local antibody concentration in the perfused segment was significantly higher than the concentration in the whole liver at the same antibody dose (Figure 3D). Histological analyses showed that increases in the DOL of the fluorescein-labeled antibody were accompanied by increases in the EC_50_ value of antibody binding (Figure 3E). However, increases in the DOL did not significantly affect capture efficacy during isolated liver perfusion (Figure 3F). Furthermore, the capture efficacy was not significantly different among antibodies labeled with different fluorophores (Figure 3G). After local antibody enrichments, the RPE-labeled antibody achieved the highest MFI ratio (Figure 3H).

### CLE-based image evaluations with clone 390 and YN1/1.7.4 mAb

**Figure 4:**
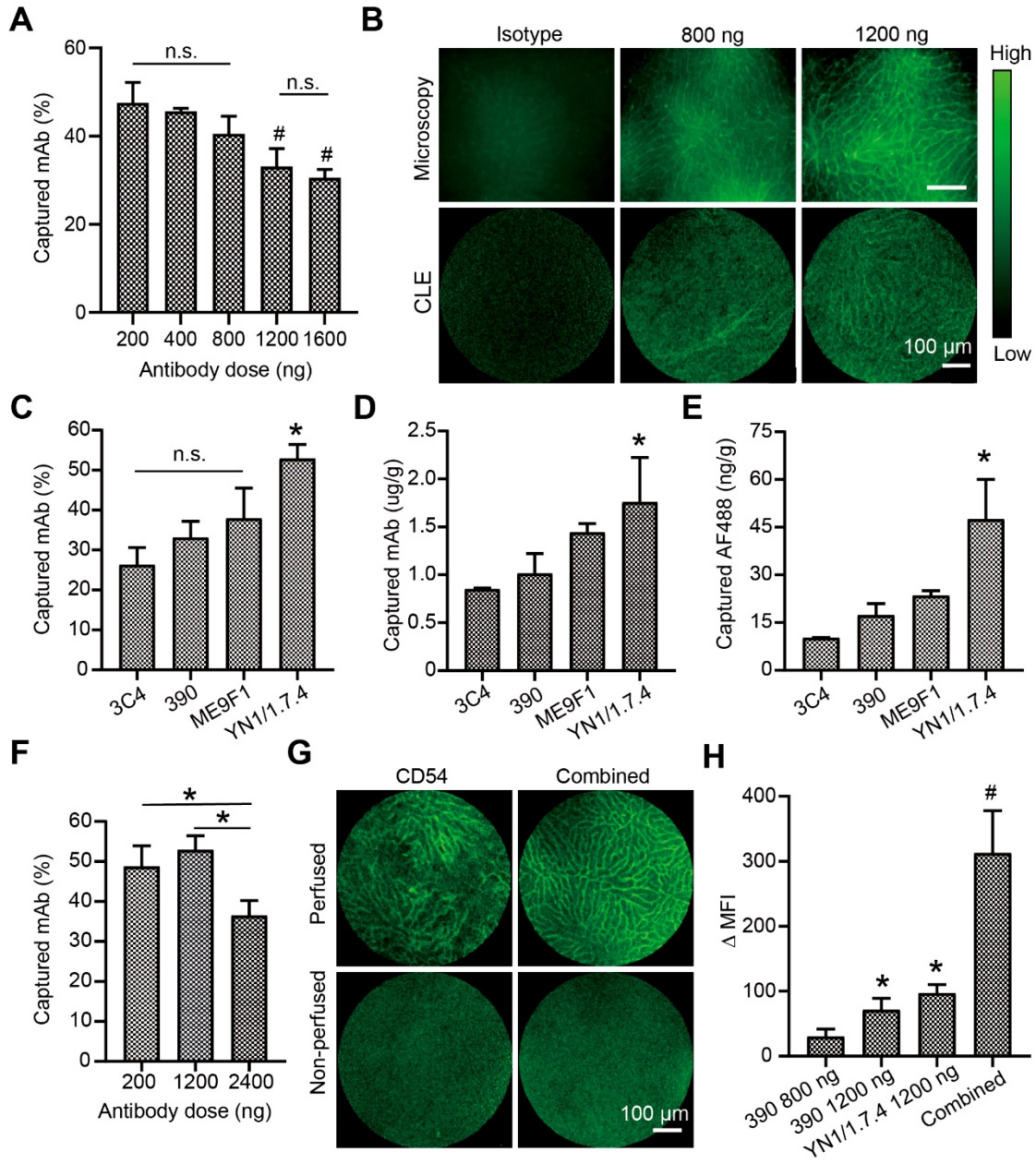
Visualization and analysis of a perfused liver segment with confocal laser endomicroscopy (CLE). (A) Dose-response analysis of endothelial antibody capture efficacy for AF488-labeled clone 390 mAb; n=3. (B) Representative images compare resolutions of conventional fluorescence microscopy and CLE. (C) Antibody capture efficacy after segment perfusion with 1200 ng of different mAbs, n=3. (D) Tissue antibody concentration and (E) local AF488 dye enrichment after perfusion segments with 1200 ng of different antibodies. (F) Antibody capture efficacy after perfusion with different doses of clone YN1/1.7.4 antibody, n=3-4. (G) Representative images of segments after perfusion 1200 ng of clone YN1/1.7.4 antibody (CD54) or the combination of clones YN1/1.7.4 and 390 (1200 ng each). (H) Quantitative analysis of the change in mean fluorescence intensity (MFI) for two antibodies at different doses, compared to 800 ng of clone 390. n.s. no significant difference. ^*^ *P*<0.05. ^#^ *P*<0.01.

Because CLE instruments are available for fluorescence imaging with excitation at 488 nm, the endocapt and imaging quality of AF488-labelled antibodies was analyzed. In the isolated liver perfusion model, increasing doses of the 390 clone mAb was accompanied by decreasing capture efficacies, with a significant difference between the highest and lowest doses (Figure 4A). Fluorescence CLE imaging was performed with local segmental perfusion. No fluorescent signal was detected at antibody doses below 800 ng. At 800 ng, the fluorescent signal was irregular and extremely weak (Figure 4B). At least 1200 ng of clone 390 mAb was required to distinguish the signal in perfused segments, which resulted in a minimal mAb concentration of 1 µg/g tissue. Different antibodies were then evaluated at the 1200-ng dose. The capture efficacy varied from 26.3% to 52.9% depending on the antibody clone (Figure 4C). The local concentration of the anti-CD54 antibody reached 1.8 µg/g tissue, which was significantly higher than the concentrations of other antibodies (Figure 4D). Due to the different DOLs, the local enrichment of AF488 dye was also analyzed. We found concentrations of 17.4 ng/g tissue for the anti-CD31 mAb and 47.5 ng/g tissue for the anti-CD54 mAb (Figure 4E). Next, the capture efficacies of low and high doses of anti-CD54 mAb was studied. The capture efficacy was similar at 200 ng and 1200 ng, but a higher dose (2400 ng) significantly decreased the capture efficacy (Figure 4F). Finally, the combined perfusion of anti-CD54 and anti-CD31 antibody was performed. We found that the fluorescent signal and the MFI were strongly improved with this antibody combination, compared to either antibody alone (Figure 4G-H).

### Surgical micronavigation and targeting the vascular boundary in liver with WF-FOM

**Figure 5:**
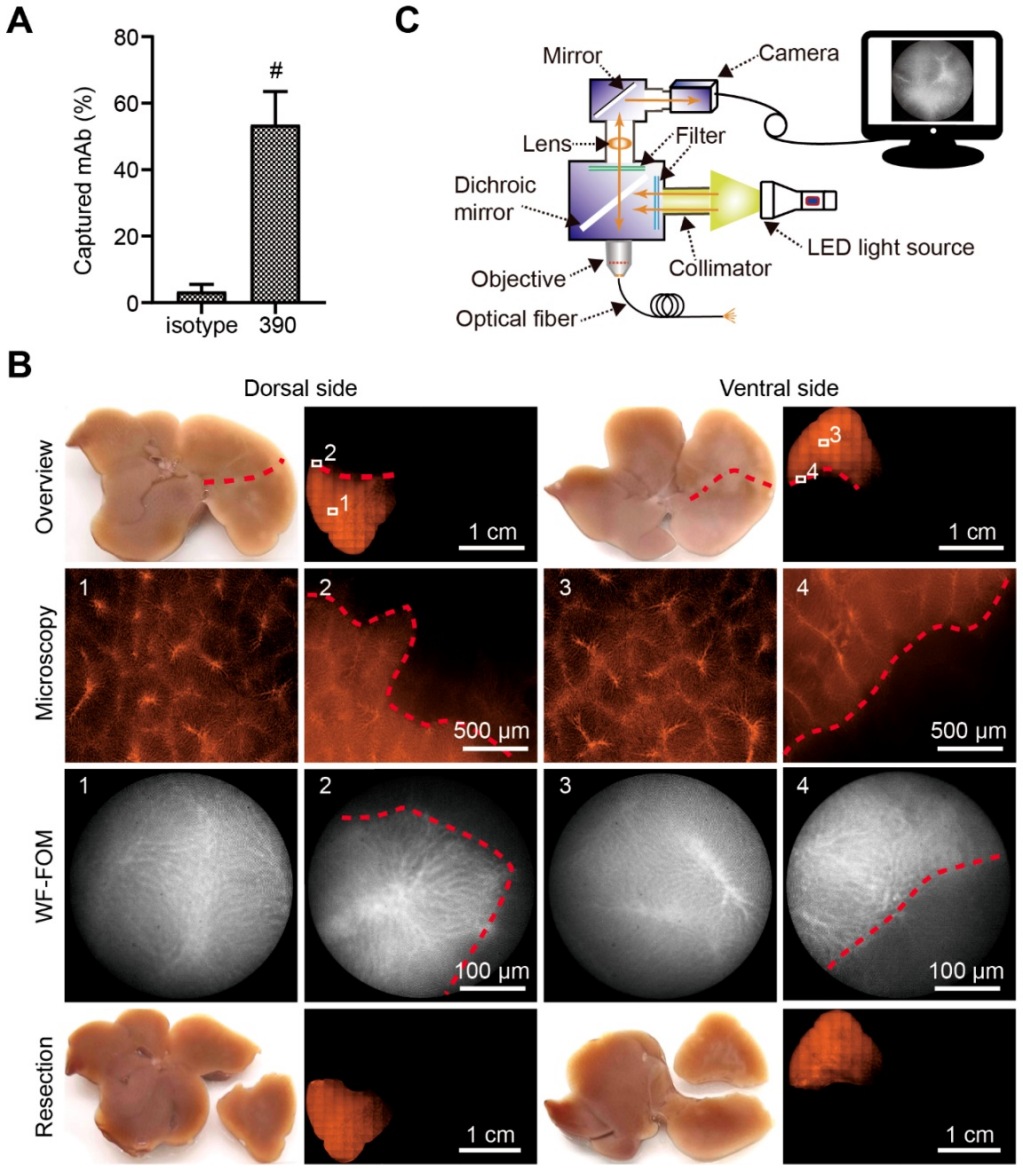
Vascular boundary identification and fluorescence-guided liver subsegment resection with FOM *ex vivo*. (A) Schematic illustration of self-assembled wide-field FOM (WF-FOM). (B) Antibody capture efficacy after perfusion with 400 ng of RPE-conjugated anti-CD31 antibody (clone 390), n=5. (C) Representative images for identification of perfused subsegment (1), boundary (2) and fluorescence-guided resection. ^#^ *P*<0.01.

After perfusion, the capture efficacy of the RPE-labeled clone 390 mAb was 53.47% (Figure 5A). This efficacy resulted in a strong fluorescence signal in the subsegment for detection with conventional fluorescence microscopy (Figure 5B). The fluorescence was additionally studied using the WF-FOM system (Figure 5C). With the WF-FOM, we detected strong fluorescent labeling in the microvascular system, even after perfusion 400 ng of clone 390 mAb. The vascular boundary of the perfused subsegment was clearly visualized with the WF-FOM, which enabled the exact dissection of the labeled subsegment. Notably, with WF-FOM guidance, we visualized the subsegment margin at both the beginning and during the dissection procedure (Figure 5B).

**Figure 6:**
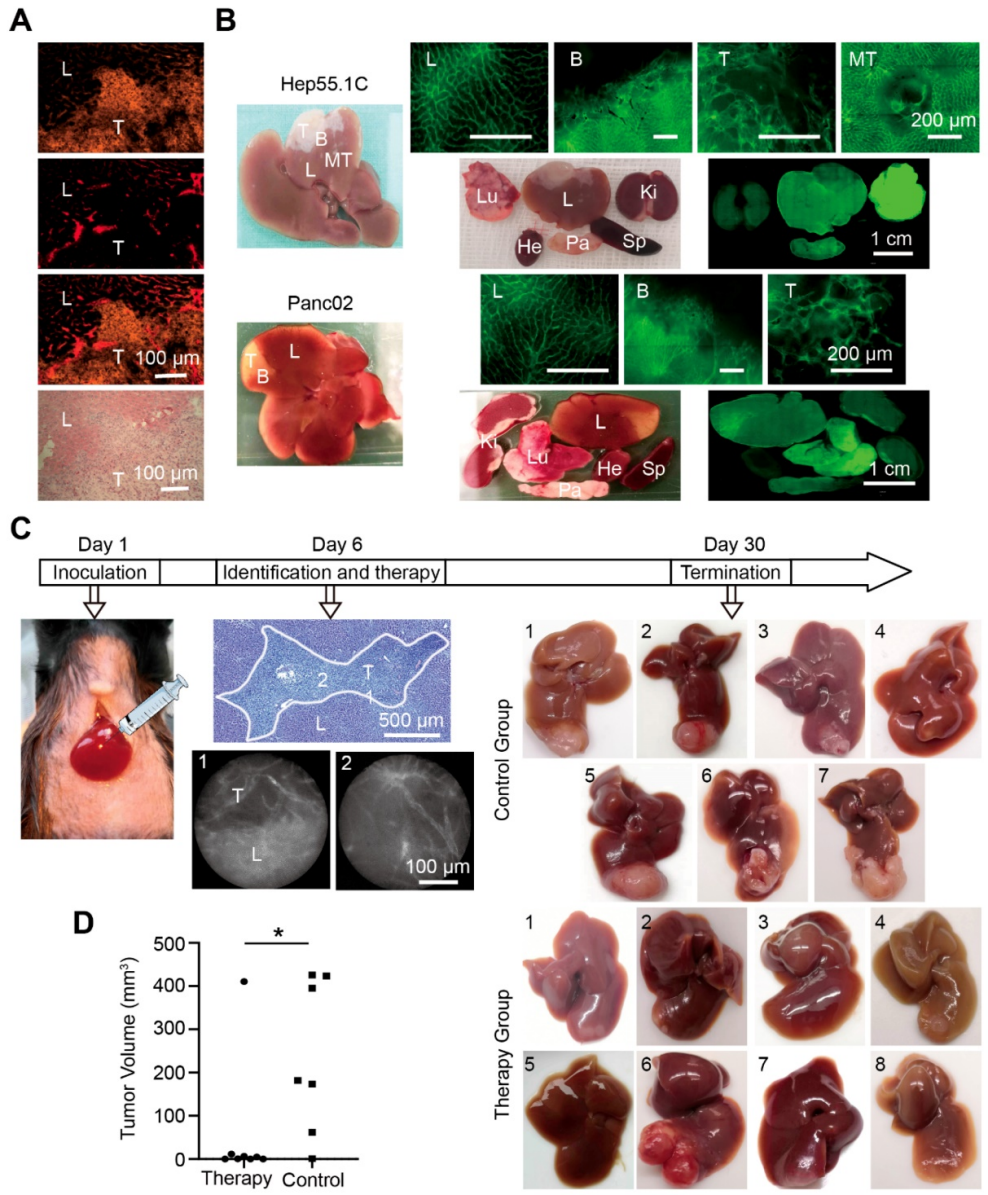
Liver tumor vascular boundary identification and boundary-target thermal ablation *in vivo*. (A) Histological tumor (T) boundary identification in liver (L) with immunofluorescence (top three panels) and H&E (bottom panel) staining. (B) Representative images of fluorescence microscopy in the liver (L), tumor (T), metastases (MT), boundary (B), the tumor bearing segment, and the organ distribution in Hep55.1C and Panc02 tumor models after perfusion with AF488-labeled clone 390 mAb. Ki, kidney; Lu, lung; Sp, spleen; Pa, pancreas; He, heart. (C) Experimental flow chart. (*Left*) Model establishment, (*middle*) fiber-optic images show *in vivo* identification of the boundary; (*right*) representative images of untreated (control) and treated (therapy) liver tumors after treatment. (D) Difference in tumor volumes between therapy and control groups.^ *^ *P*<0.05.

Immunofluorescence staining of the microvascular system showed distinct differences in microvascular angioarchitecture between normal and tumor tissues. The tumor boundary was clearly distinguished, and it was also confirmed with hematoxylin and eosin (H&E) staining (Figure 6A). To identify the liver tumor boundary* in vivo*, whole liver labeling was performed in two different mouse liver tumor models. Tumor-bearing hepatic segments accumulated the fluorescent signal in both models, and the vascular boundary was clearly detected with fluorescence microscopy. A weaker fluorescent signal was detected in the other organs except the lung, which displayed a strong signal (Figure 6B).

For therapeutic studies, we induced hepatic micro-metastatic tumors of pancreatic cancer in mice (Figure 6C). After a systemic injection of RPE-labeled clone 390 mAbs, tumors and tumor margins were identified with WF-FOM. Differences in microvascular angioarchitecture allowed the clear identification and thermo-ablation of micro-tumors (Figure 6C). WF-FOM-guided treatment resulted in complete tumor elimination in 3 mice, and nearly complete tumor elimination (>90% of the mean size reduction) in 4 mice (Figure 6D). One mouse in the treatment group had a large tumor remnant after therapy (Figure 6C, mouse #6). At the end of the *in vivo* experiments, we confirmed the presence or absence of tumors with histological H&E staining.

## Discussion

In the present study, we investigated the efficacy of FOM for identifying the vascular boundary of tumors after labeling endothelial cells with fluorescent antibodies. A comparative analysis of antibody binding showed that all antibodies, except the anti-CD34 mAbs, were detectable in both human and mouse hepatic sinusoidal and tumor endothelial cells. This finding was consistent with findings in previous studies 31, and it confirmed that cell surface endothelial cell markers were well conserved in mice and humans 32. It should be noted that the heat-map data provided quantitative, background-corrected values of mean fluorescence, but these values depended strongly on the fraction of blood vessels that expressed the antigen and on the density of local blood vessels. These parameters could only be estimated with direct visualization. For example, both clone 581 and Qbend-10 showed high MFIs in liver; however, they only labeled a fraction of the blood vessels.

Each antibody clone has a characteristic epitope specificity and an individual binding affinity 33. The endothelial cell surface is directly exposed to circulating blood, which facilitates antibody binding in the setting of intravascular injections 22. Single antibody clones must rapidly bind to the antigen on endothelial cells for effective capture during short exposures 29. As previously shown, endocapt in living cell cultures could provide information about epitope location (extracellular or intracellular) and facilitated histological studies 22. In the present study, we showed that cultured endothelial cells could capture selected antibody clones* in vitro*, which demonstrated the availability of extracellular epitopes for endocapt. Furthermore, endothelial antigens can also be expressed by other cells, for example by specialized leukocyte subpopulations (CD31, CD54, CD102) 34-37 or some tumor cells (CD54) 38, 39. In the current study, the endothelial capture of intravascular injected antibodies is a dominating process.

The cellular antigen density and the rate of antibody internalization might determine the quality of antibody-based imaging 20. In the present study, we found that the half-lives of selected anti-human clones was at least 6 h, which would be sufficient for continuous intraoperative imaging with navigation. We also showed that none of the selected clones were cytotoxic to endothelial cells *in vitro*. However, further toxicological studies are necessary prior to potential pre-clinical evaluations.

Conjugation can affect the antibody binding properties 40. It was shown that fluorescein coupling to a mouse monoclonal anti-HA antibody (Fc125) had a minor effect on avidity, but a significant fraction of antibody was inactivated at higher conjugation levels 41. Our results were consistent with that study. Increasing the DOL of the fluorescein-labeled antibody to 8.5 resulted in a 2.5-fold reduction in binding activity. However, despite this reduced binding ability, the capture efficacy remained high (up to 50%) at high DOLs. Thus, the reduction in binding did not directly lead to a suppression of endocapt efficacy and might depend on individual fluorophores. As previously shown, we found that conjugating the antibody with a large fluorophore, such as RPE, suppressed endocapt efficacy although it remained sufficient for targeting aims 22.

CLE systems are available with cellular resolution 42, 43, which can facilitate microcirculation imaging 44. For excitation, the CLE used a high-intensity laser. However, the very short exposure times required sufficient local fluorophore concentrations. In the present study, the CLE sensitivity was lower than that of conventional fluorescence microscopy and of WF-FOM. Imaging blood vessels with CLE in mouse liver required a local concentration of at least 17 ng AF488/g tissue.

As described above, it is necessary to accurately determine the extent of resection of segments or subsegments during anatomical resection 4, 45. Our previous study showed the technical ability for labeling of tumor-bearing liver segments after superselective injection of anti-CD146 mAb in mice in vivo 46 and using intravascular leukocyte sequestration *ex vivo* 47. In the current study, we performed liver subsegment identification following microvascular labeling. We showed that labeling the subsegmental microvascular system and observing with WF-FOM provided excellent accuracy in identifying the margin. Furthermore, the segmental margin could be discriminated continuously during liver dissections, which facilitated exact liver resections. This approach provided significant advantages over current ICG-based methods, where image-guided navigation is difficult during dissection 48.

In the current study, we showed that the tumor margins in experimental tumors could be readily identified with microvascular labeling and WF-FOM. We also showed that WF-FOM-guided thermo-ablation was very effective for the local control of nearly all (7 of 8) treated tumors. Only one tumor was probably not correctly identified, and it showed progressive growth after the treatment. The identification of microscopic liver tumors is an important clinical problem. Although the liver resection is normally aimed to remove completely the tumor tissue and to achieve the tumor-free (R0) status, the significant percentage of operations is accompanied by remaining of microscopic tumors (R1-resection). It varies between 8 and 46% for colorectal metastases and has negative consequences on patient survival 49, 50. Our findings suggested that WF-FOM-guided identification of microvascular tumor margins could potentially be useful for the *in situ* recognition and destruction or elimination of microscopic tumors in human liver (e.g., tumor remnants after R1 resections). This important issue must be investigated in further studies.

Microvascular labeling should be performed with FDA-approved ramucirumab or other selected clones. As shown in Figure S2A-B, the vascular boundary could be clearly determined using immunofluorescence (Figure S2A) and immunohistochemistry (Figure S2B) on human histological sections. For local endocapt-based antibody enrichment in human, the superselective injection using intraportal (for liver segment imaging) or intraarterial (for both tumor and liver segment imaging) could be proposed (depicted in the illustration Figure S2C). However, extensive toxicological and metabolic studies are required for all new, non-approved antibodies prior to use in patients. It must also be taken into the attention that the lung is the first organ which capture antibody after intravenous injection before its distribution in the whole body. As demonstrated in the current study, it can result in high intrapulmonary antibody enrichment in vivo. This finding confirms results of our previous studies 22 and corresponds well with results of other authors 51, 52.

In summary, the present study revealed the basic principles of vascular boundary identification at the microscopic level with fluorescent endothelial labeling and FOM. We propose that this approach could facilitate at least two potential clinical applications for surgical micronavigation: segment border identification during liver dissections and the identification of tumor margins.

## Supplementary Material

Supplementary figures and tables.

## Figures and Tables

**Table 1:** List of primary and secondary antibodies.

Anti-mouse antibodies				Anti-human antibodies			
Antigen	Clone	Conjugation	Source	Antigen	Clone	Conjugation	Source
CD34	SA376A4	RPE	Biolegend	CD34	581	RPE/Pure	Biolegend
CD34	MEC14.7	RPE	Biolegend	CD34	Qbend-10	RPE/Pure	Exbio
CD34	RAM34	RPE	BD Biosciences	CD31	JC70/A	Pure	Abcam
CD34	HM34	RPE/Pure	Biolegend	CD31	WM59	RPE/Pure/AF488	Biolegend
CD31	390	RPE/Pure/AF488/AF647	Biolegend	CD31	MEM05	RPE/Pure	Exbio
CD31	MEC13.3	RPE	Biolegend	CD31	1D2-1A5	Pure	Abnova
CD54	YN1/1.7.4	RPE/Pure/AF488	Biolegend	CD54	HA58	RPE/Pure	Biolegend
CD146	ME9F1	RPE/AF488	Biolegend	CD146	SHM-57	RPE/Pure	Biolegend
CD105	MJ7/18	RPE/AF488	Biolegend	CD105	43A3	RPE/Pure	Biolegend
CD102	3C4	RPE	SouthernBiotech	CD102	CBR-IC2/2	RPE/Pure	Biolegend
CD102	3C4	AF488	Biolegend	Ctrl	MOPC-21	RPE/Pure	Biolegend
Ctrl	RTK2758	RPE/AF488/AF647	Biolegend	Ctrl	MOPC-173	RPE/Pure	Biolegend
Ctrl	RTK4530	RPE/AF488	Biolegend	Goat anti-mouse	Poly4053	RPE	Biolegend

Source specification: Biolegend (San Diego, CA, USA), BD Biosciences (Heidelberg, Germany), SouthernBiotech (Birminham, AL, USA), Exbio (Praque, Czech Republik), Abnova (Taipeh, Taiwan), Abcam (Cambridge, UK)

## Abbreviations

HCC: hepatocellular carcinoma
ICG: Indocyanine green
endocapt: endothelial capture
CLE: confocal laser endomicroscopy
FOM: fiber-optic microscopy
RPE: R-phycoerythrin
AF: Alexa Fluor
WF-FOM: wide-field fiber-optic microscopy
MFI: mean fluorescence intensity
EC_50_: half-maximal effective concentration
Crc MTS: liver metastases from colorectal cancer
LMTS: liver metastases from pancreatic cancer
HUVEC: human umbilical vein endothelial cells
HDMEC: human dermal microvascular endothelial cells
DOL: degree of labeling
mAb: monoclonal antibody
H&E: hematoxylin and eosin
PBS: phosphate-buffered saline
