# Supplementary material for: Fluorescence-guided fiber-optic micronavigation using microscopic identification of vascular boundary of liver segment and tumors
Source: Theranostics. 2020 May 15;10(14):6136–48. doi: 10.7150/thno.45973 (PMC7255018; doi:10.7150/thno.45973)

Supplemental Figure S1. Representative immunofluorescence images of endothelium-specific antibody binding in human (A, B) and mouse (C, D) tissue. HCC, hepatocellular carcinoma; NP, normal pancreas; LMTS, liver metastasis of pancreatic cancer.

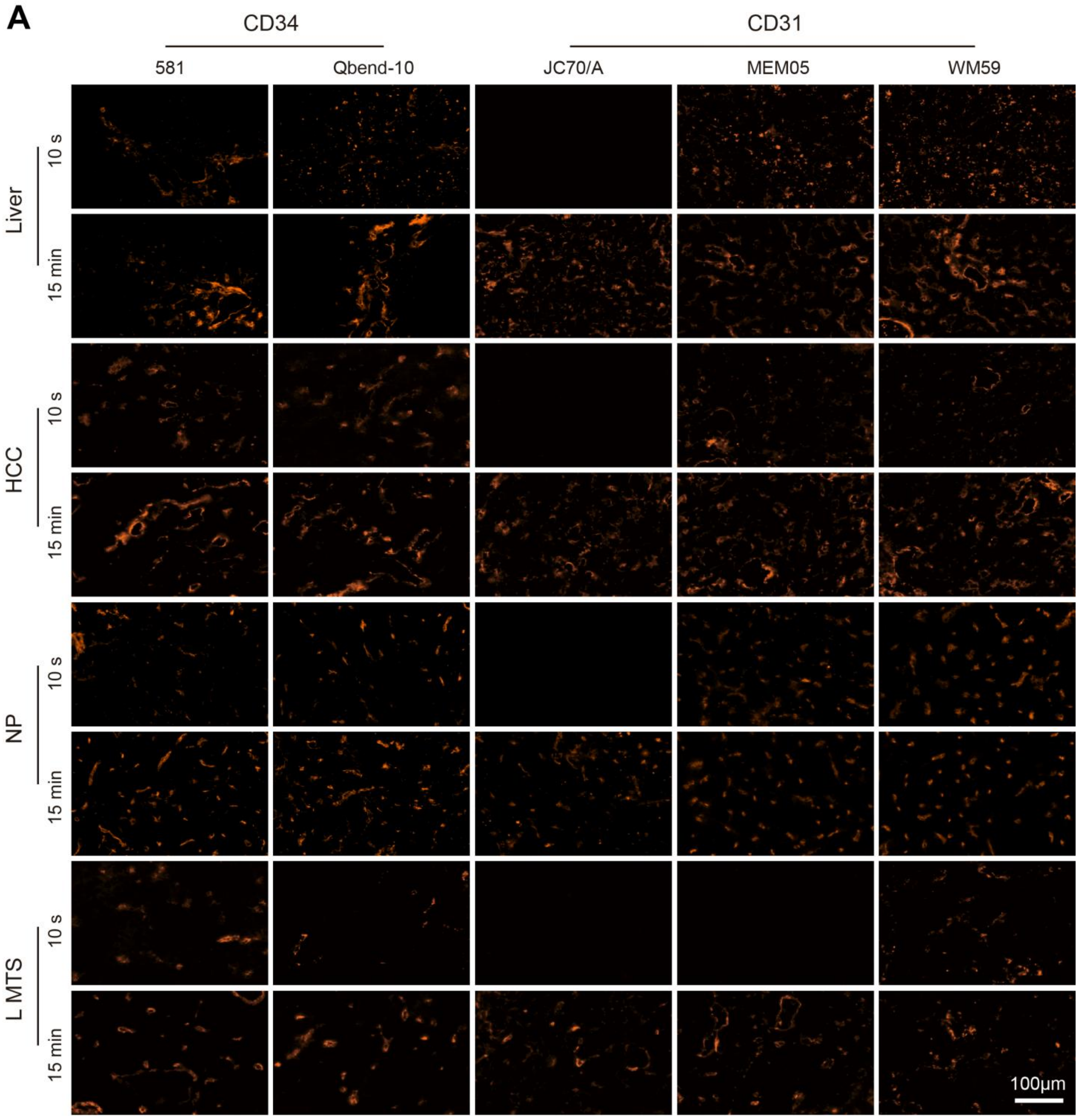

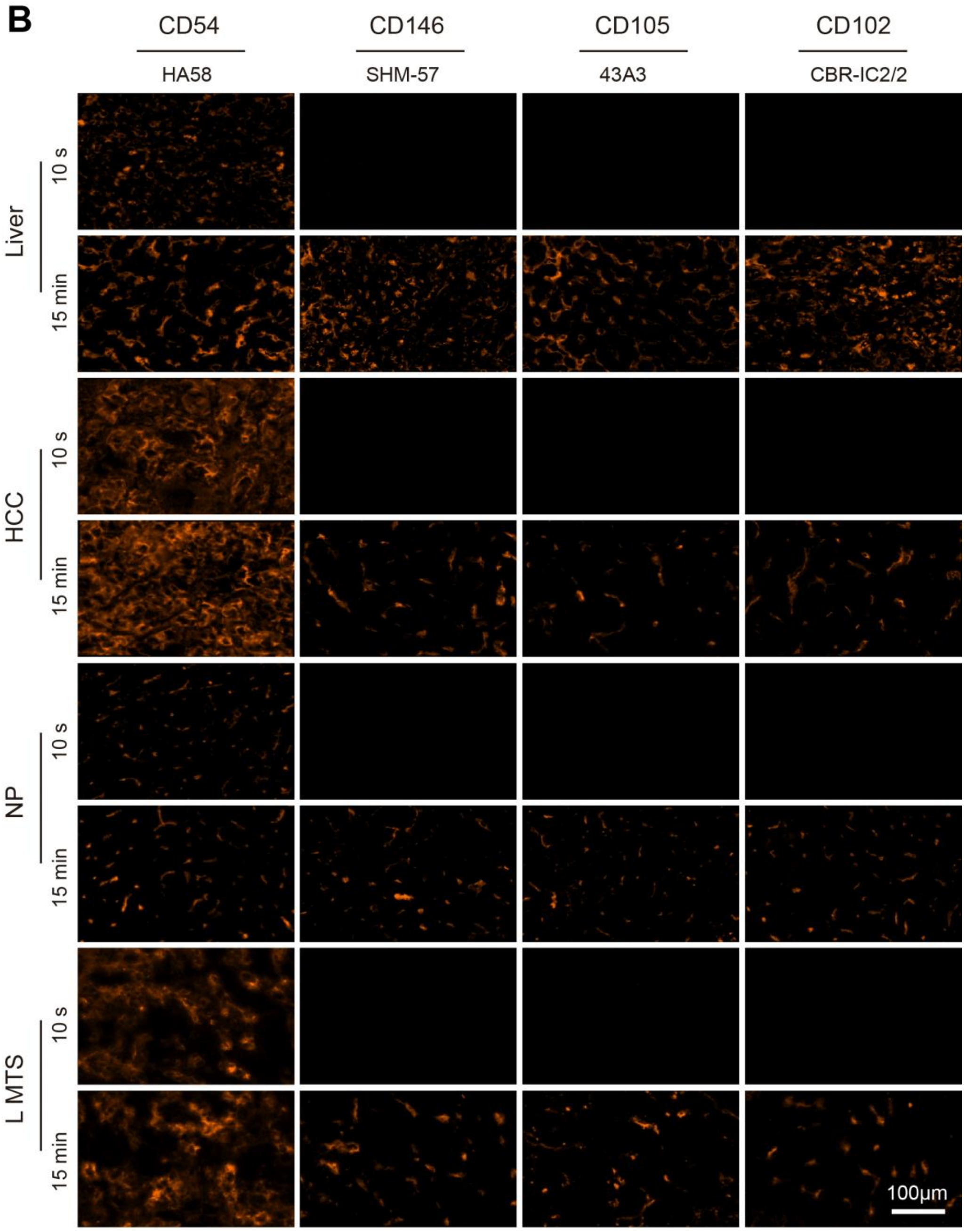
B
CD54
HA58
CD146
SHM-57
CD105
43A3
CD102
CBR-IC2/2
Liver
HCC
NP
L MTS
10 s
15 min
100μm

C

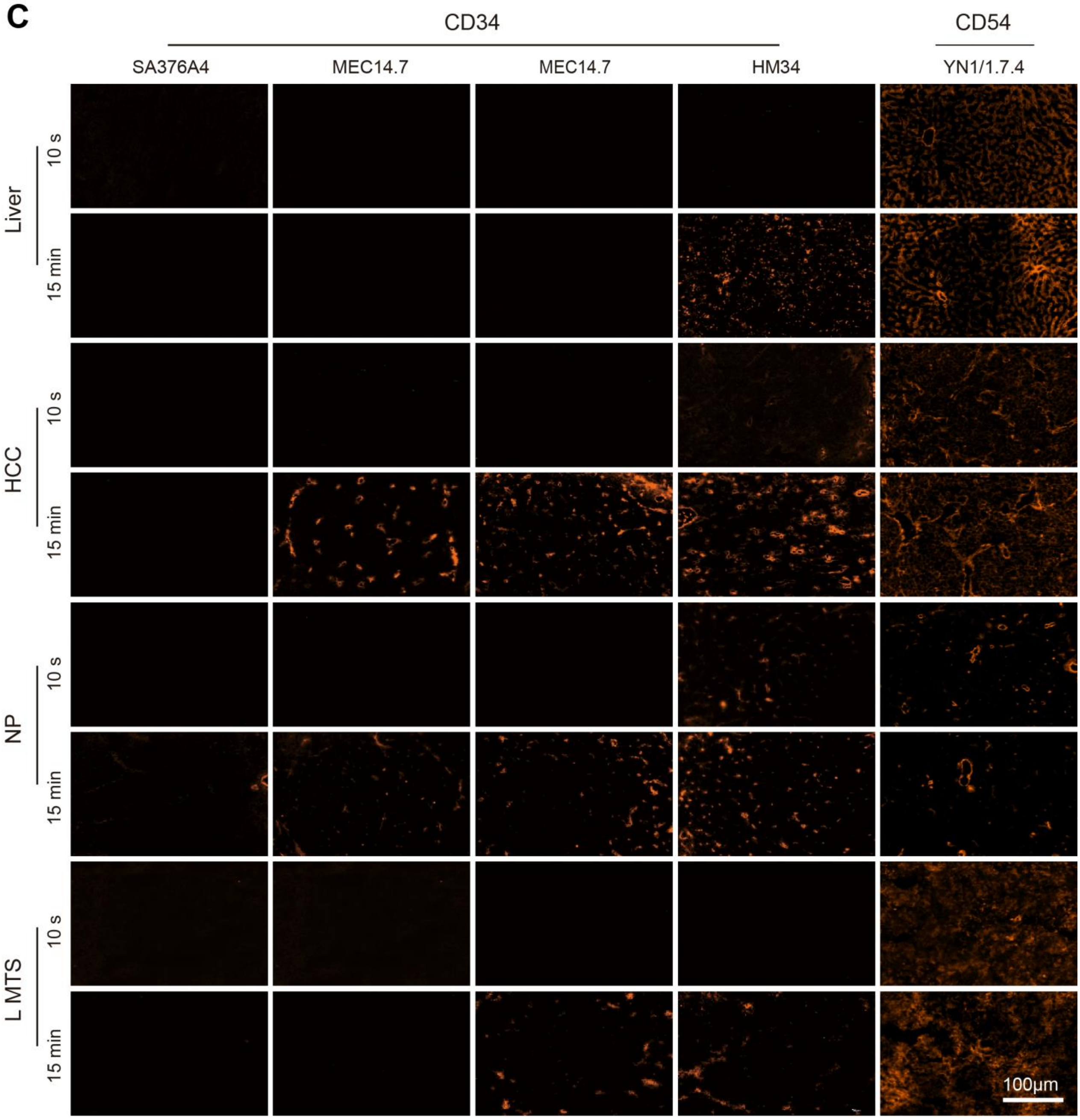

CD34
CD54
SA376A4
MEC14.7
MEC14.7
HM34
YN1/1.7.4
Liver
10 s
15 min
HCC
10 s
15 min
NP
10 s
15 min
L MTS
10 s
15 min
100μm

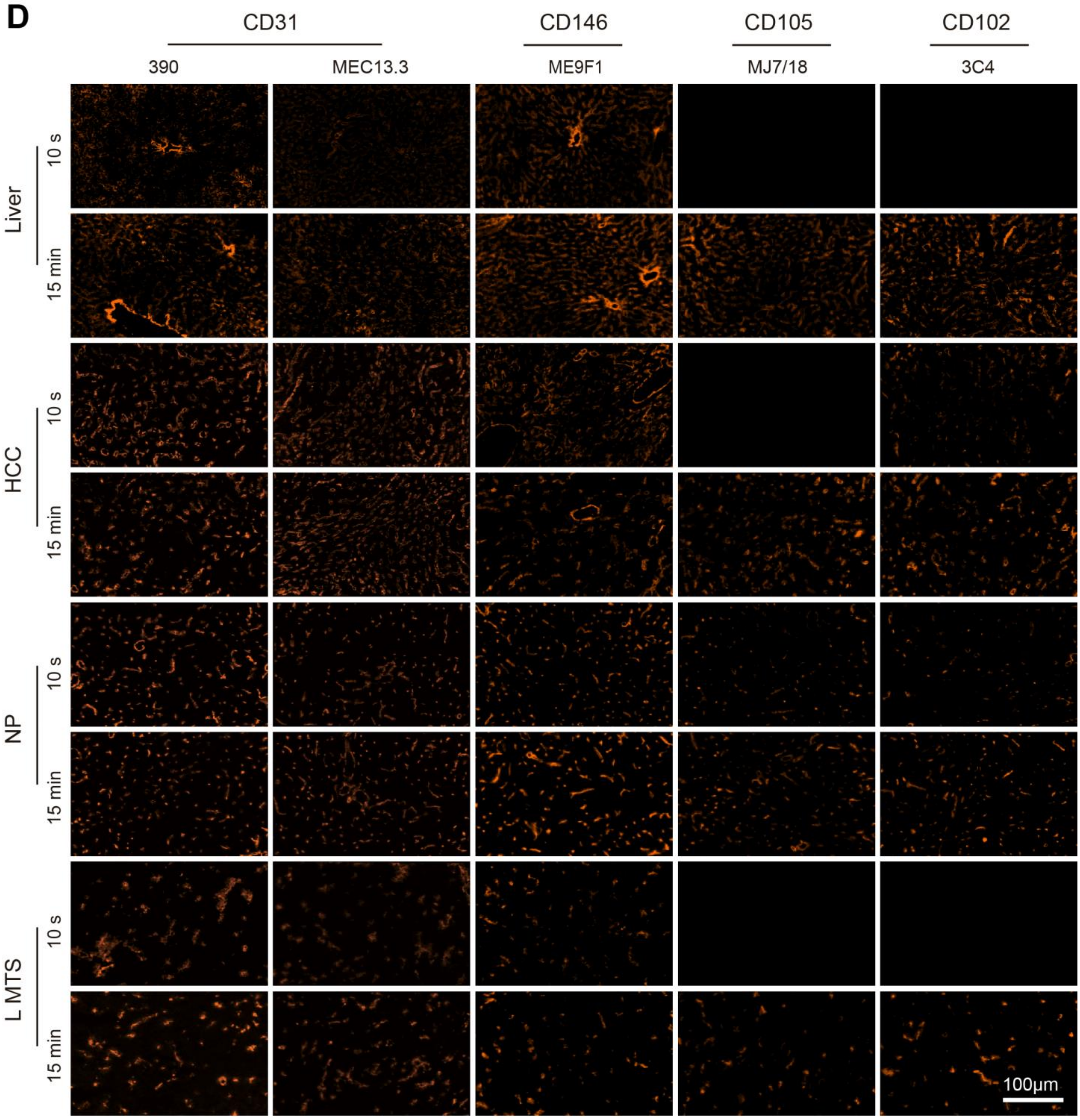
D
CD31
CD146
CD105
CD102
390
MEC13.3
ME9F1
MJ7/18
3C4
Liver
HCC
NP
L MTS
10 s
15 min
100μm

Supplemental Figure S2. Histological identification of vascular boundaries using selected anti-human mAb clones and the illustration of the superselective injection procedure. (A) Double immunofluorescence staining of a liver metastasis of pancreatic cancer with antibody clones WM59 and HA58. (B) Representative images of CD34 expression in different human liver tumors. HCC: hepatocellular carcinoma; LMTS, liver metastasis of pancreatic cancer; Crc MTS: Liver metastasis of colorectal cancer (C) Illustration of the principle of the endothelial capture technique in human applications. The superselective injection of antibodies could be achieved using intraportal (for liver segment imaging, left picture) or intraarterial (for tumor and liver segment imaging, right picture) access.

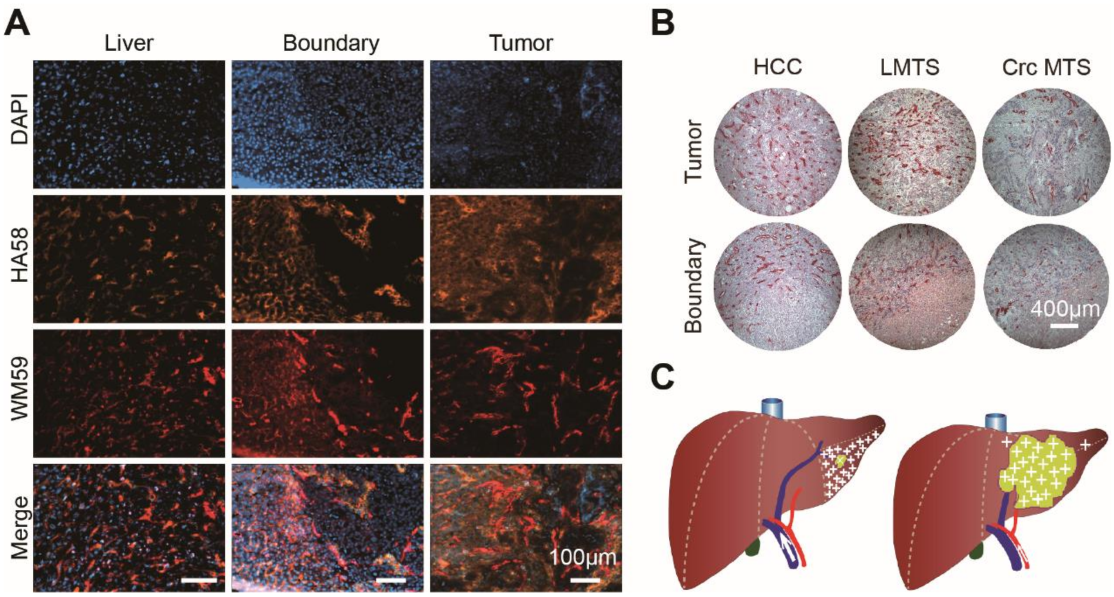

Supplement: Supplementary file 1 — Supplementary figures and tables. [file thnov10p6136s1.pdf]
